# Preliminary data on the psychometric proprieties of the italian version of the reflective functioning questionnaire

**DOI:** 10.1192/j.eurpsy.2021.1362

**Published:** 2021-08-13

**Authors:** P. Velotti, G. Rogier, S. Beomonte Zobel, E. Ciavolino, S. Beyer, P. Fonagy

**Affiliations:** 1 Dynamical And Clinical Psychology, Sapienza Università di Roma, Rome, Italy; 2 Dipartimento Di Storia, Società E Studi Sull`uomo, Università del Salento, Lecce, Italy; 3 Division Of Psychology And Language Sciences, University College London, London, United Kingdom

**Keywords:** RFQ, italian validation, Reflective functioning, psychometric properties

## Abstract

**Introduction:**

Assessing mentalizing abilities is a complex issue. Only recently an instrument assessing mentalizing capacity as a whole, the Reflective Functioning Questionnaire (RFQ), has been developed.

**Objectives:**

To reach the purpose of our study, we investigated the psychometric proprieties of the Italian version of the RFQ.

**Methods:**

The study was conducted on a sample including a group of violent offenders and a group of community participants. All subjects fulfilled the RFQ, the Personality Inventory for DSM-5 (PID-5) and the Aggression Questionnaire (AQ).

**Results:**

The theoretical model was defined and analysed by using Partial Least Squares–Path Modelling with high-order construct definition. Data showed good psychometric proprieties of the Italian version of the RFQ. Also, specific patterns of correlations were identified between the RFQ subscales and both PID-5 and AQ scores. Offenders significantly differed from controls only in relation to one subscale of the RFQ.

**Conclusions:**

Data supported the factorial structure of the RFQ found in the original validation study. Results also support the existence of a second-order variable, mentalizing, resulting from the convergence of hypomentalizing and hypermentalizing.

